# Integrated analysis identifies GABRB3 as a biomarker in prostate cancer

**DOI:** 10.1186/s12920-024-01816-8

**Published:** 2024-01-29

**Authors:** Jun-Yan Chen, Chi-Fen Chang, Shu-Pin Huang, Chao-Yuan Huang, Chia-Cheng Yu, Victor C. Lin, Jiun-Hung Geng, Chia-Yang Li, Te-Ling Lu, Bo-Ying Bao

**Affiliations:** 1 Department of Pharmacy, China Medical University, 100 Jingmao Road Section 1, 406 Taichung, Taiwan; 2 Department of Anatomy, School of Medicine, China Medical University, 406 Taichung, Taiwan; 3 Department of Urology, Kaohsiung Medical University Hospital, 807 Kaohsiung, Taiwan; 4 Graduate Institute of Clinical Medicine, College of Medicine, Kaohsiung Medical University, 807 Kaohsiung, Taiwan; 5 Institute of Medical Science and Technology, College of Medicine , National Sun Yat-Sen University, 804 Kaohsiung, Taiwan; 6 Department of Urology, College of Medicine, National Taiwan University Hospital, National Taiwan University, 100 Taipei, Taiwan; 7 Division of Urology, Department of Surgery, Kaohsiung Veterans General Hospital, 813 Kaohsiung, Taiwan; 8 Department of Urology, School of Medicine, National Yang Ming Chiao Tung University , 112 Taipei, Taiwan; 9 Department of Pharmacy, Tajen University, 907 Pingtung, Taiwan; 10 Department of Urology, E-Da Hospital, 824 Kaohsiung, Taiwan; 11 School of Medicine for International Students, I-Shou University, 840 Kaohsiung, Taiwan; 12 Department of Urology, Kaohsiung Municipal Hsiao-Kang Hospital, 812 Kaohsiung, Taiwan; 13 Graduate Institute of Medicine, College of Medicine, Kaohsiung Medical University, 807 Kaohsiung, Taiwan; 14 Department of Medical Research, Kaohsiung Medical University Hospital, 807 Kaohsiung, Taiwan

**Keywords:** PI3K/AKT pathway, GABRB3, Prostate cancer, Survival, Gene set enrichment analysis

## Abstract

**Background:**

Treatment failure following androgen deprivation therapy (ADT) presents a significant challenge in the management of advanced prostate cancer. Thus, understanding the genetic factors influencing this process could facilitate the development of personalized treatments and innovative therapeutic strategies. The phosphoinositide 3-kinase (PI3K)/AKT signaling pathway plays a pivotal role in controlling cell growth and tumorigenesis. We hypothesized that genetic variants within this pathway may affect the clinical outcomes of patients undergoing ADT for prostate cancer.

**Methods:**

We genotyped 399 single-nucleotide polymorphisms (SNPs) across 28 core PI3K/AKT pathway genes in a cohort of 630 patients with prostate cancer undergoing ADT. We assessed the potential association of the SNPs with patient survival. Functional analyses of the implicated genes were also performed to evaluate their effects on prostate cancer.

**Results:**

After multivariate Cox regression analysis and multiple testing correction, *GABRB3* rs12591845 exhibited the most significant association with both overall and cancer-specific survivals (*P* < 0.003). A comprehensive pooled analysis of 16 independent gene expression datasets revealed elevated expression of *GABRB3* in prostate cancer tissues compared to that in normal tissues (*P* < 0.001). Furthermore, gene set enrichment analysis unveiled differential enrichment of pathways such as myogenesis, interferon γ and α responses, and the MYC proto-oncogene pathway in tumors with elevated *GABRB3* expression, implying a role for *GABRB3* in prostate cancer.

**Conclusion:**

Our results suggest that rs12591845 could potentially serve as a valuable prognostic indicator for patients undergoing ADT. The potential role of *GABRB3* in promoting prostate tumorigenesis is also highlighted.

## Introduction

Prostate cancer is a significant health concern and the leading cause of cancer-related fatalities in men, accounting for over 1.4 million new diagnoses and approximately 375,304 associated deaths in 2020 [1]. Understanding the association of prostate cancer with androgens has paved the way for androgen deprivation therapy (ADT) [2], the primary treatment for locally advanced and metastatic cases. However, extended ADT often leads to prostate cancer recurrence, marked by increased prostate-specific antigen (PSA) levels and the emergence of castration-resistant prostate cancer (CRPC), which adversely affects prognosis [3]. Alarmingly, statistical analyses have revealed that over 84% of patients are diagnosed with metastases at the onset of CRPC, and the median survival following CRPC diagnosis is only 14 months [4]. Therefore, discovery of prognostic markers that can identify high-risk patients is crucial. Additionally, improved interventions are necessary to extend survival in patients with prostate cancer.

The mechanisms underlying ADT resistance largely involve androgen-related factors, such as amplified androgen biosynthesis, adrenal androgens, and disruptions in androgen receptor (AR) signaling [5]. Intriguingly, disturbances in AR signaling can induce the activation of secondary signaling cascades, notably the phosphoinositide 3-kinase (PI3K)/serine/threonine kinase (AKT) pathway, which may play a pivotal role in CRPC development [5, 6]. In transgenic mouse models, suppression of AR signaling resulted in heightened PI3K/AKT activity, driven by reduced levels of PH domain and leucine rich repeat protein phosphatases (PHLPPs), a suppressor of AKT, due to downregulation of the AR target gene FKBP prolyl isomerase 5 (FKBP5) [5, 6]. Elevated PI3K/AKT signaling has been observed in a significant proportion of patients with prostate cancer, positively correlating with CRPC, hastened tumor progression, and enhanced invasiveness [7, 8]. Immunohistochemical analysis revealed robust phospho-AKT (Ser473) staining in over 90% of cancer tissues with Gleason score of 8–10, compared to only 10% in tissues with lower Gleason scores [9]. Distinct AKT isoforms exhibit variable effects on tumorigenesis and cell migration, with AKT1 suppressing cancer cell migration and AKT2 promoting it [10, 11]. The regulatory subunits (p85) of PI3K also exhibited significant roles; p85α (PIK3R1) acted as a tumor suppressor with reduced expression in cancer, whereas p85β (PIK3R2) functioned as an oncogene, showing heightened expression in cancers [12]. Mutations in PI3K catalytic subunit genes, especially p110α (PIK3CA), were frequently identified in prostate cancer with concurrent PIK3CA mutation and loss of phosphatase and tensin homolog (PTEN) accelerating tumorigenesis and promoting CRPC in mouse models [13].

Recent studies have explored the relationship between single nucleotide polymorphisms (SNPs) in the PI3K/AKT pathway and susceptibility to prostate cancer. Among the analyzed SNPs, the *mechanistic target of rapamycin kinase* (*MTOR*) rs17036508, *MTOR* rs2295080, *regulatory associated protein of MTOR complex 1* (*RPTOR*) rs1468033, and *AKT2* rs7250897 are associated with prostate cancer risk in Chinese men [14]. Additionally, the *MDM2 proto-oncogene* (*MDM2*) rs2279744 influenced prostate cancer risk and survival across different subtypes of tumor protein *P53* rs1042522 carriers, whereas *MDM2* rs2279744 alone did not have a significant association with prostate cancer outcomes [15–17]. However, it remains unclear whether genetic variants in the PI3K/AKT pathway have a prognostic value for prostate cancer progression after ADT.

In this study, we examined the correlation between 399 SNPs in 28 genes linked to the PI3K/AKT pathway and the survival outcomes in a cohort of 630 patients with advanced prostate cancer who underwent ADT. Furthermore, we explored the biological functions of the implicated gene, gamma-aminobutyric acid type A receptor subunit beta 3 (GABRB3), to uncover plausible biological mechanisms affecting the progression of prostate cancer.

## Methods

### Patient response evaluation

In this study, 630 patients diagnosed with prostate cancer underwent ADT at three medical centers: National Taiwan University Hospital, Kaohsiung Medical University Hospital, and Kaohsiung Veterans General Hospital, were recruited. The study was approved by the Institutional Review Board of Kaohsiung Medical University Hospital (KMU-HIRB-2013132) and followed Good Clinical Practice guidelines. Written informed consent was obtained from all the participants. Relevant clinicopathological data were extracted from the medical records. Progression-free survival (PFS) refers to the duration from treatment initiation to the first occurrence of biochemical, local, regional, or nodal failures, distant metastasis, or death from cancer. Overall Survival (OS) refers to the duration from treatment initiation to death from any disease. Cancer-specific survival (CSS) refers to the duration from treatment initiation to death from prostate cancer. Additional clinicopathological information regarding this study is available in previous publications [18, 19]. Of the 630 patients, 414 died, with 314 succumbing to prostate cancer over a median follow-up period of 150.8 months [20]. Clinical factors such as age, PSA level at ADT initiation, clinical stage, Gleason score at diagnosis, PSA nadir, and time to PSA nadir were significantly associated with both OS and CSS (*P* < 0.05).

### SNP selection and genotyping

Haplotype-tagged SNPs across 28 major genes related to the PI3K/AKT pathway were identified. These genes encompassed *AKT1*, *AKT2*, *AKT3*, *ataxin 1* (*ATXN1*), *coiled-coil domain containing 88 A* (*CCDC88A*), *DEP domain containing MTOR interacting protein* (*DEPTOR*), *GABRB1*, *GABRB2*, *GABRB3*, *huntingtin* (*HTT*), *MTOR associated protein LST8 homolog* (*MLST8*), *MTOR*, *paladin* (*PALLD*), *pyruvate dehydrogenase kinase 1* (*PDK1*), *PHLPP1*, *PHLPP2*, *phosphatidylinositol-4,5-bisphosphate 3-kinase catalytic subunit alpha*, *beta*, and *delta* (*PIK3CA*, *PIK3CB*, and *PIK3CD*), *phosphoinositide-3-kinase regulatory subunit 1–2* (*PIK3R1-2*), *PTEN*, *protein phosphatase 2 phosphatase activator* (*PTPA*), *RPTOR independent companion of MTOR complex 2* (*RICTOR*), *ribosomal protein S6 kinase B1* (*RPS6KB1*), *RPTOR*, and *TSC complex subunit 1–2* (*TSC1-2*). The selection utilized the Haploview 4.2 tagger algorithm, employing 1000 Genomes Project data from Han Chinese individuals in Beijing and Southern Han Chinese individuals [21]. Genomic DNA was isolated from peripheral lymphocytes and genotyped using the Affymetrix Axiom Genotyping Array system (Thermo Fisher Scientific, Waltham, MA, USA) at Taiwan’s National Centre for Genome Medicine, following established protocols [22]. SNPs that did not meet the following criteria were excluded: genotyping call rates under 0.9, minor allele frequencies below 0.05, and deviations from Hardy–Weinberg equilibrium exceeding 0.001. As a result, 399 haplotype-tagged SNPs were retained for further analysis.

### Bioinformatic analysis

To explore *GABRB3* expression differences between prostate cancer and normal tissues, we utilized gene expression datasets from the ArrayExpress (E-MEXP-1327 and E-TABM-26), Gene Expression Omnibus (GSE14206, GSE17951, GSE21032, GSE26910, GSE30174, GSE30521, GSE30522, GSE32448, GSE3325, GSE6919, GSE6956, GSE7307, and GSE8218), and The Cancer Genome Atlas Prostate Adenocarcinoma (TCGA PRAD, https://portal.gdc.cancer.gov/) databases. A pooled analysis was performed using Review Manager(Cochrane, London, UK), employing a random-effects model to address potential heterogeneity across studies. To delve further into the molecular mechanisms and associated pathways of *GABRB3*, we conducted gene set enrichment analysis (GSEA)-based gene ontology and hallmark pathway investigations using GSEA software. We categorized 497 TCGA PRAD samples into high (upper quartile) and low (lower quartile) groups based on *GABRB3* expression levels. Gene ontology (GO) and hallmark gene sets in the Human Molecular Signatures Database were used as reference genes. The normalized enrichment score was obtained through gene set permutations with 1000 times, and the significance was set at false discovery rate *q*-values [23] < 0.05, as the enrichment threshold.

### Statistical analysis

All statistical analyses were performed using Statistical Product and Service Solutions version 20.0.0(IBM, Armonk, NY, USA). Statistical significance was determined at a two-sided *P*-value of less than 0.05. Univariate and multivariate Cox regression analyses were performed to elucidate the relationships between genotypes, clinical variables, and patient prognosis. To assess the impact of genotype on OS and CSS in patients with prostate cancer, Kaplan-Meier survival analysis and the log-rank test were employed. Additionally, *q*-values were computed to substantiate meaningful associations identified in the study.

## Results

**Fig. 1:**
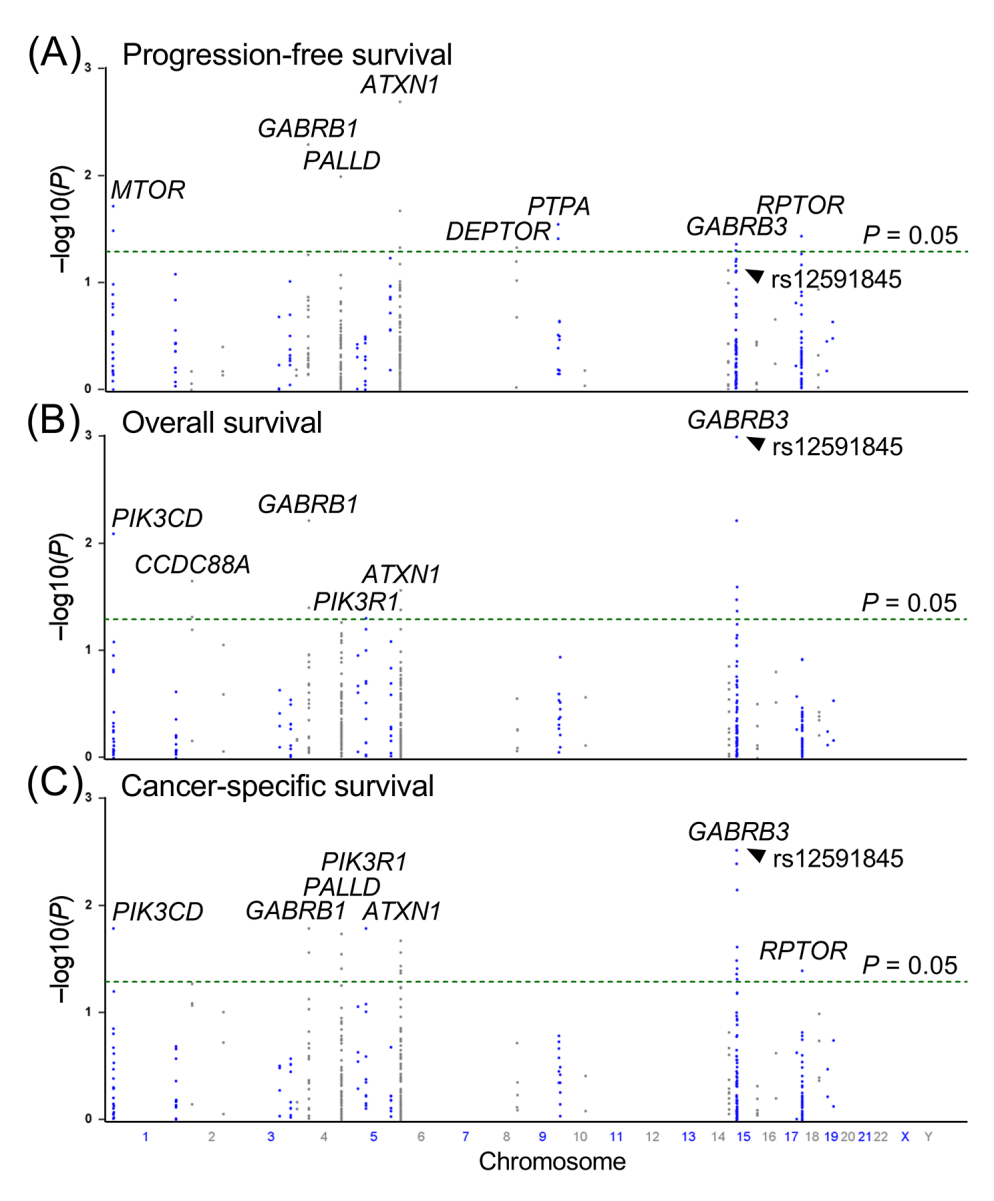
Manhattan plots depict the relationships (Y-axis as − log10(*P*) values) between 399 single-nucleotide polymorphisms (X-axis as their positions on chromosomes) located within 28 PI3K/AKT pathway genes and progression-free survival **(A)**, overall survival **(B)**, and cancer-specific survival **(C)** of patients with prostate cancer undergoing androgen-deprivation therapy. The nominal significance threshold (*P* = 0.05) is represented by a dashed horizontal line and genes exhibiting noteworthy relationships are indicated

**Fig. 2:**
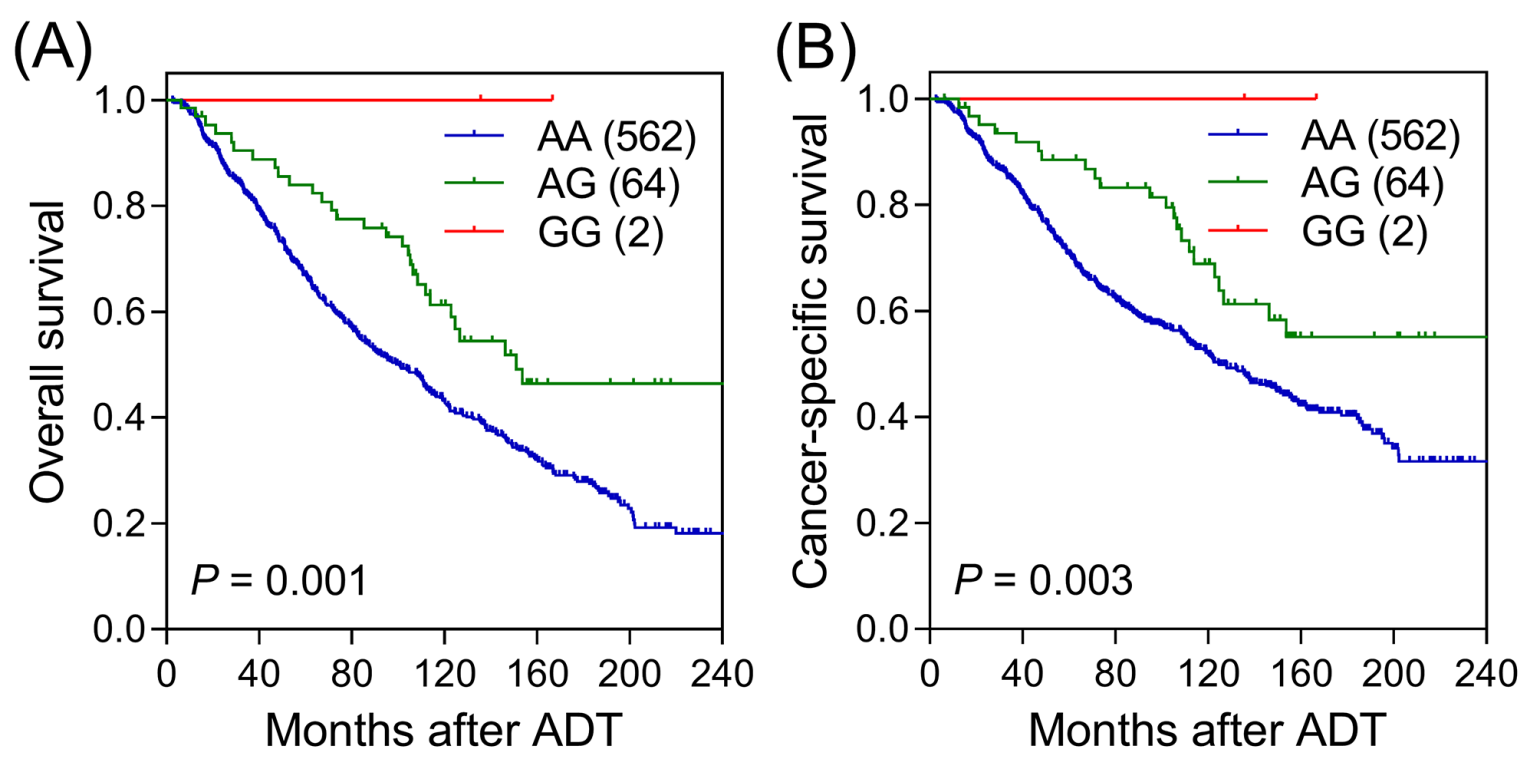
Kaplan–Meier estimation displays overall survival **(A)** and cancer-specific survival **(B)** among patients with prostate cancer categorized based on the genotypes of *GABRB3* rs12591845. The values in parentheses indicate the patient count within each subgroup

To investigate the potential connection between PI3K/AKT signaling and prostate cancer progression, we analyzed the relationship between 399 SNPs in 28 genes associated with the PI3K/AKT pathway and survival outcomes after ADT. Among the examined SNPs, 15 variants (two in *MTOR*, one in *GABRB1*, two in *PALLD*, four in *ATXN1*, one in *DEPTOR*, two in *PTPA*, two in *GABRB3*, and one in *RPTOR*) were associated with PFS (*P* < 0.05; Fig. 1A), 13 SNPs (one in *PIK3CD*, two in *CCDC88A*, two in *GABRB1*, one in *PIK3R1*, two in *ATXN1*, and five in *GABRB3*) were associated with OS (Fig. 1B), and 22 SNPs (one in *PIK3CD*, two in *GABRB1*, three in *PALLD*, one in *PIK3R1*, six in *ATXN1*, eight in *GABRB3*, and one in *RPTOR*) were associated with CSS (Fig. 1C). Notably, the most prominent signal was identified in *GABRB3* rs12591845, which displayed significant associations with both OS (*P* = 0.001, with a *q*-value of 0.374; Table 1) and CSS (*P* = 0.003) but exhibited only a weak association with PFS (*P* = 0.078). Specifically, each additional minor allele G of rs12591845 was linked to a 45% reduction in the risk of all-cause mortality (hazard ratio [HR] = 0.55, with a 95% confidence interval [CI] = 0.38–0.79, Table 1; Fig. 2A) and a 47% reduction in the risk of cancer-specific mortality (HR = 0.53, 95% CI = 0.35–0.81, Table 1; Fig. 2B). To evaluate the impact of *GABRB3* rs12591845 beyond the scope of clinical variables on survival outcomes, multivariate analysis was performed, adjusting for established predictors such as age, PSA at ADT initiation, clinical stage, Gleason score at diagnosis, PSA nadir, and time to PSA nadir. Even after accounting for these clinical factors, the significance of *GABRB3* rs12591845 persisted for both OS (*P* = 0.001) and CSS (*P* = 0.007; Table 1).

**Table 1:** Association of *GABRB3* rs12591845 with OS and CSS in patients receiving ADT

Genotype	n of patients	OS					CSS				
		10-year OS	HR (95% CI)	***P***	HR (95% CI)^a^	***P*** ^a^	10-year CSS	HR (95% CI)	***P***	HR (95% CI)^a^	***P*** ^a^
AA	562	43.2	1.00		1.00		52.1	1.00		1.00	
AG	64	61.3	0.58 (0.40–0.83)	0.003	0.53 (0.36–0.79)	0.002	68.9	0.55 (0.36–0.86)	0.008	0.57 (0.36–0.89)	0.013
GG	2	100.0	-	-	-	-	100.0	-	-	-	-
AG/GG	66	62.6	0.55 (0.38–0.80)	0.002	0.52 (0.35–0.77)	0.001	70.1	0.53 (0.35–0.82)	0.004	0.55 (0.35–0.86)	0.009
Trend^b^			0.55 (0.38–0.79)	0.001	0.52 (0.35–0.76)	0.001		0.53 (0.35–0.81)	0.003	0.55 (0.35–0.85)	0.007

Abbreviations: OS: overall survival; CSS: cancer-specific survival; ADT: androgen deprivation therapy; HR: hazard ratio; CI: confidence interval

^a^ Adjustment for age, PSA at ADT initiation, clinical stage, Gleason score at diagnosis, PSA nadir, and time to PSA nadir

^b^ The trend *P*-values were calculated using a linear trend model with a continuous variable for the number of rare G alleles

-: not calculated due to small sample size

**Fig. 3:**
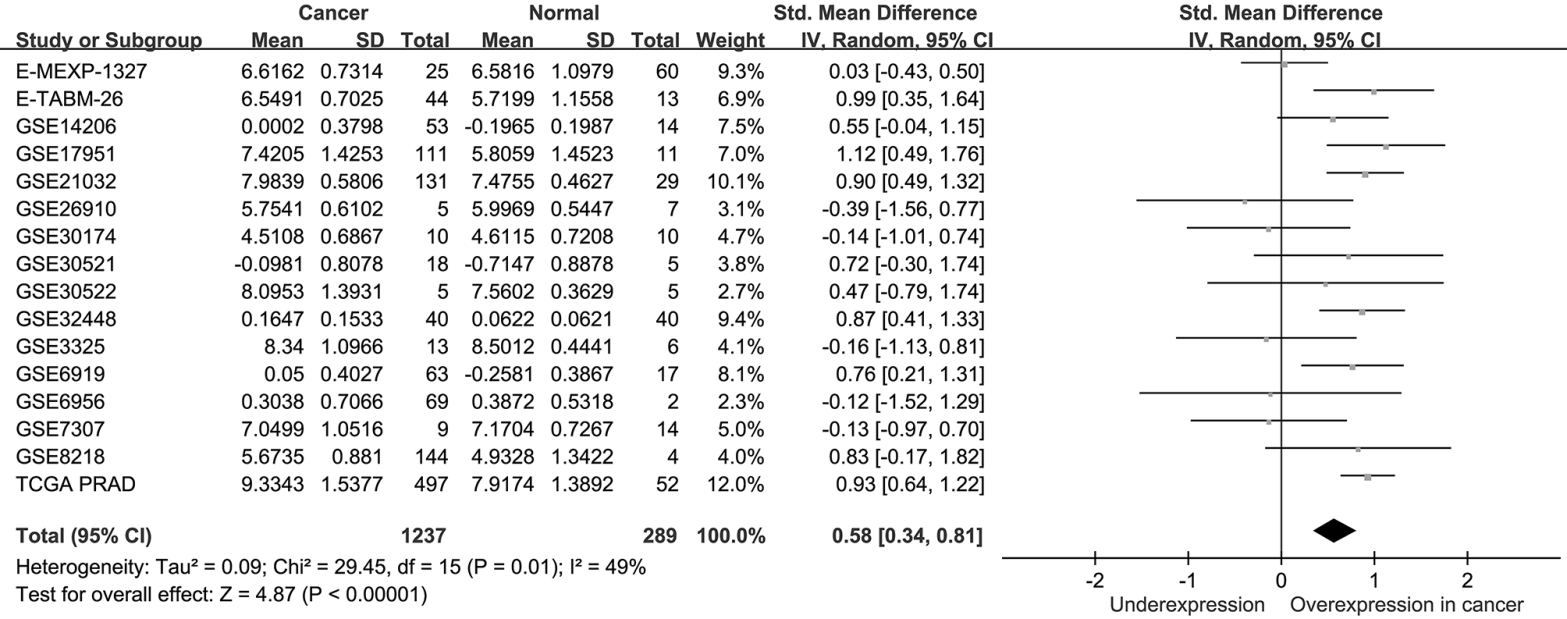
A pooled analysis of 16 studies demonstrated elevated expression of *GABRB3* in prostate cancer tissues compared to normal tissues. TCGA PRAD: The Cancer Genome Atlas prostate adenocarcinoma; SD: standard deviation; IV: inverse variance; CI: confidence interval; Std: standardized; df: degrees of freedom

To understand the clinical implications of *GABRB3* in prostate cancer, we conducted a pooled analysis of *GABRB3* expression levels between prostate cancer and the corresponding normal tissues by employing 16 publicly accessible prostate cancer gene expression datasets with 1237 prostate cancer samples and 289 normal prostate tissues. The results indicated a notably higher expression of *GABRB3* in prostate cancer compared to normal prostate tissues (standardized mean difference = 0.58, 95% CI = 0.34–0.81, *P* < 0.001, Fig. 3).

**Fig. 4:**
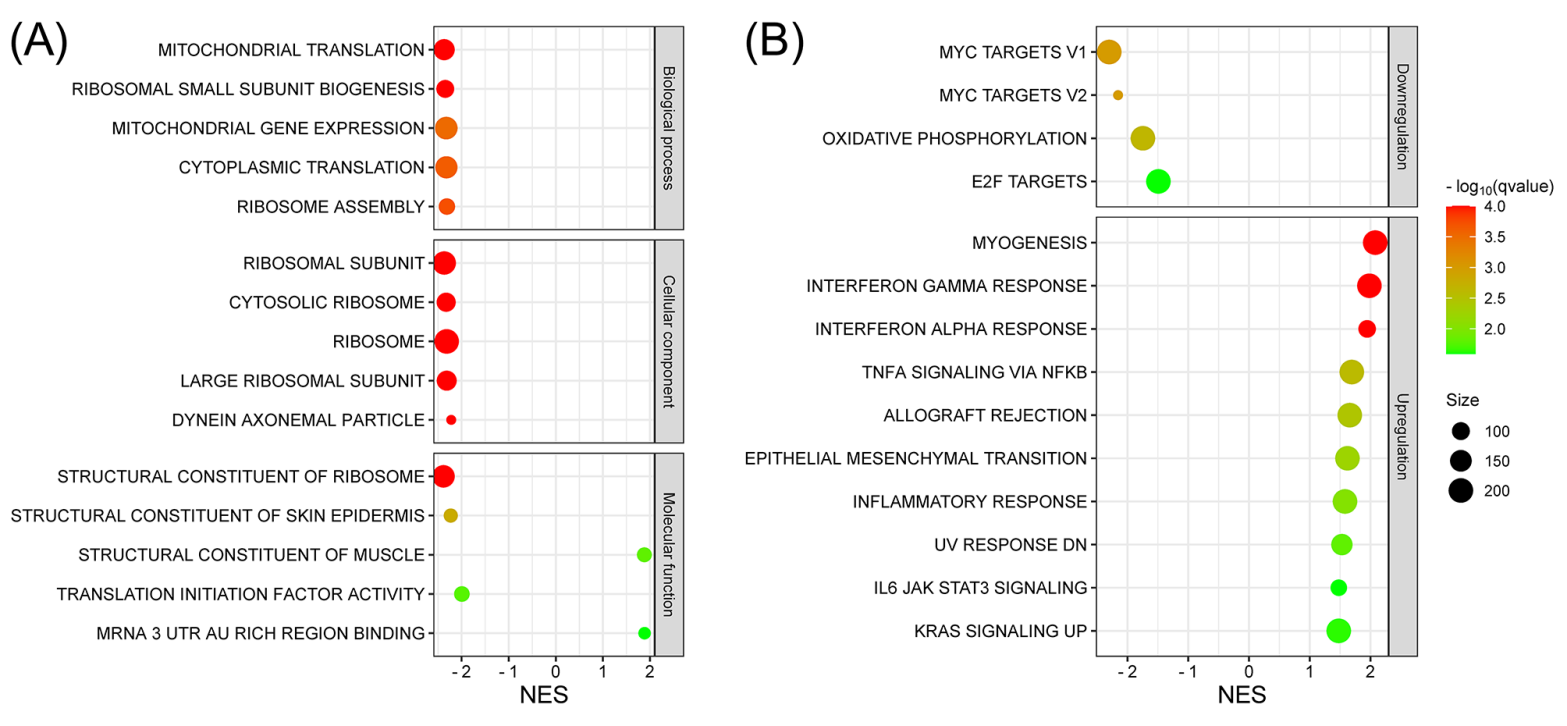
Gene set enrichment analysis for gene ontology (GO) and hallmark pathways between tumors with high and low *GABRB3* expression. **(A)** The five most prominent terms related to biological processes, cellular components, and molecular functions in GO enrichment analysis. **(B)** Enriched hallmark pathway gene sets that were upregulated or downregulated in tumors with high *GABRB3* expression. NES, normalized enrichment score

To further elucidate the molecular mechanisms underlying the roles of *GABRB3* in prostate cancer, we performed GSEA on prostate cancer tumors exhibiting high and low *GABRB3* expression using the GO and Hallmark pathway databases. The top five GO categories are shown in Fig. 4A. In terms of biological processes, the most significantly enriched categories were those associated with the downregulation of mitochondrial translation, ribosomal small subunit biogenesis, and mitochondrial gene expression. In the cellular component category, the downregulated genes were predominantly linked to ribosomes and ribosomal subunits. Furthermore, molecular function analysis revealed that the downregulated genes primarily encoded structural constituents of the ribosome and skin epidermis. Conversely, the upregulated genes were enriched in the structural constituents of muscle. Subsequent utilization of the Hallmark pathway database demonstrated significant downregulation (*q* < 0.05) of four gene sets and significant upregulation of ten gene sets in high-*GABRB3* expression tumors (Fig. 4B). The pathways showing the highest degrees of enrichment in the high-*GABRB3* expression samples were myogenesis, interferon gamma (IFNG) response, and interferon alpha (IFNA) response. Additionally, two pathways, MYC targets v1 and v2, were downregulated.

## Discussion

**Fig. 5:**
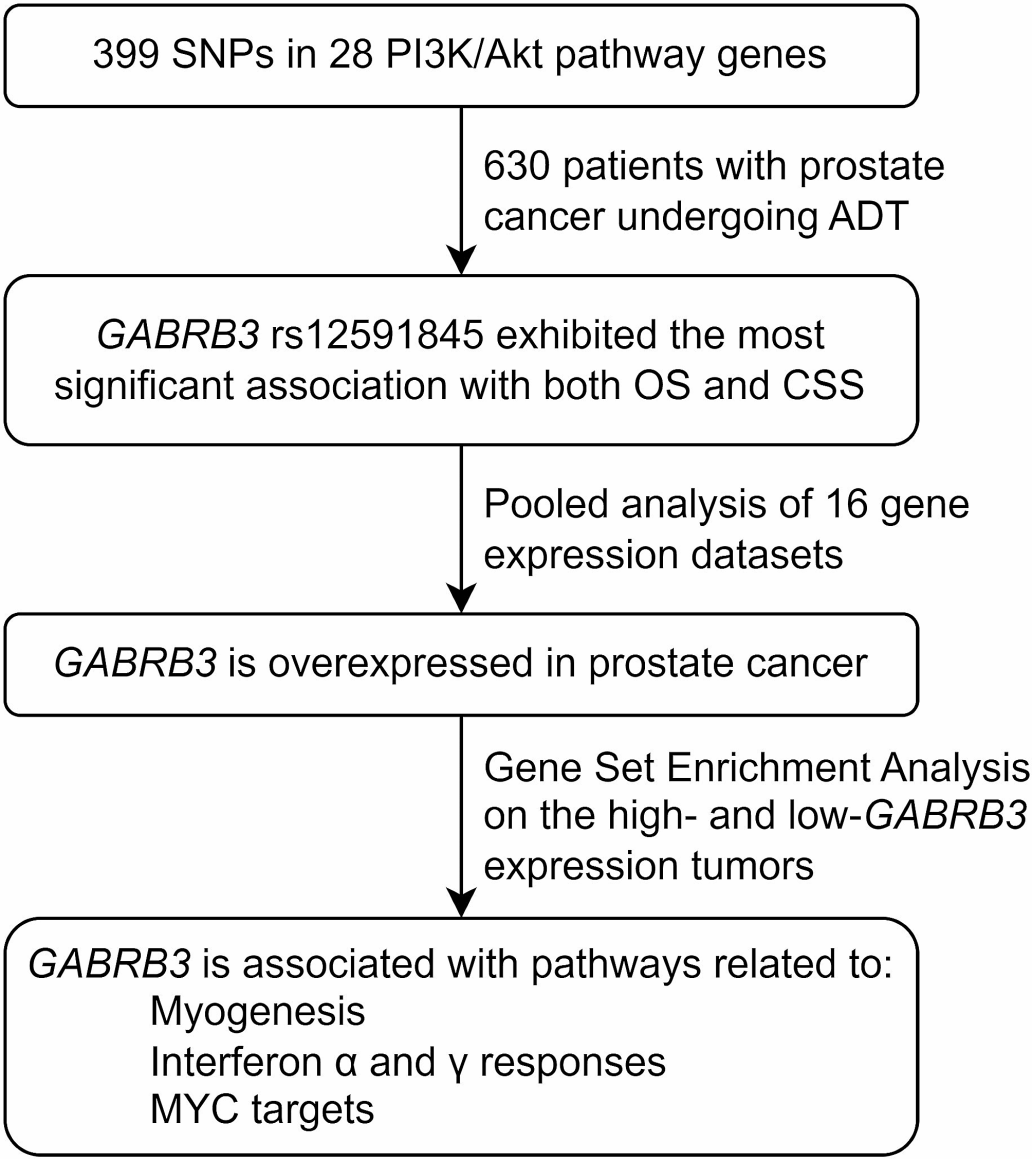
Study design and key findings. SNP: single-nucleotide polymorphism; PI3K: phosphoinositide 3-kinase; ADT: androgen deprivation therapy; OS: overall survival; CSS: cancer-specific survival

Prostate cancer prognosis is influenced by various factors, including stage, Gleason score, PSA levels, and their dynamics. Even among patients with relatively similar clinical features and treatments, there can be wide variations in prognosis. Genetic predisposition has been proposed as a contributing factor to this variability [24]. As illustrated in Fig. 5, we assessed the association between PI3K/AKT pathway gene SNPs and survival outcomes in patients undergoing ADT for prostate cancer in the current study. Our findings revealed that *GABRB3* rs12591845 is independently associated with both OS and CSS. Additionally, a pooled analysis of 16 independent studies demonstrated that *GABRB3* mRNA expression levels in prostate tumor tissue specimens were significantly higher than those in adjacent non-cancerous samples. To gain further insight into the role of *GABRB3* in prostate cancer, GSEA revealed that tumors with high *GABRB3* expression were enriched in several cancer-related pathways. These results suggest the potential involvement of *GABRB3* in the development and progression of prostate cancer.

The strongest signal, tagged with rs12591845, resided within the *GABRB3* intron. Functional annotations from RegulomeDB [25] and HaploReg [26] bioinformatics tools revealed that rs12591845 coincides with chromatin accessibility peaks in the brain and blood vessel cells, along with alterations in the binding motifs of several transcription factors, including EBF transcription factor 1, glucocorticoid receptor, myocyte enhancer factor 2, and PLAG1 zinc finger protein. These observations suggest a potential role for rs12591845 in the modulation *GABRB3* expression. However, it is noteworthy that expression quantitative trait loci analysis was unavailable in both the genotype-tissue expression [27] and 1000 Genomes databases [21], likely because of the extremely low minor allele frequency in non-East Asian populations (less than 1% in Africans and Europeans compared with 5% in East Asians). We further explored proxy untyped SNPs exhibiting high linkage disequilibrium with the risk variant. Our investigation revealed that a proxy untyped SNP, rs28666077, showing substantial linkage disequilibrium (D’=1.0) with rs12591845, exhibited a correlation between the major allele and elevated *GABRB3* expression levels in prostate tissue samples compared to the minor allele (*P* = 0.017). These findings suggest that the rs12591845 risk allele A may potentially increase *GABRB3* expression, which in turn is associated with tumor development and patient prognosis.

*GABRB3* encodes the β3-subunit of the gamma-aminobutyric acid type A receptor (GABAAR), a key player in inhibitory postsynaptic potential in the nervous system mediated by gamma-aminobutyric acid (GABA). Intriguingly, evidence suggests that GABAAR plays a role in prostate cancer progression during ADT [28, 29]. Notably, increased expression of GABA synthetic enzyme glutamate decarboxylase 1 was observed in NCI-H660, PC-3, and DU145 CRPC cell lines. Interestingly, no detectable expression was observed in LNCaP and LAPC4 androgen-responsive cell lines [28]. Furthermore, GABA elevation in neuroendocrine-like cells induces the secretion of gastrin-releasing peptides (GRP), promoting the invasive potential of PC-3 prostate cancer cells [29]. Additionally, GABA and GABAAR agonists, such as isoguvacine, were found to enhance epidermal growth factor receptor signaling, thereby promoting the proliferation of PC-3 and LNCaP prostate cancer cells [30]. These findings suggest a potential link between GABA signaling and prostate cancer progression.

Transcriptomic analysis is a promising approach to gain a comprehensive understanding of underlying molecular mechanisms. In this study, we performed a transcriptomic analysis to investigate the mechanisms associated with *GABRB3* in human prostate cancer. Hallmark pathway analysis revealed the enrichment of 14 signaling pathways in tumors with high and low *GABRB3* expression levels. Several of these pathways corroborate prior findings. For instance, MYC downregulation has been previously reported as a survival strategy for cancer cells under conditions of limited energy resources [31]. MYC protein levels decrease in cancer cells that are distant from blood vessels, and RNA interference-mediated MYC downregulation decreases necrotic cell death induced by oxygen and glucose deprivation. Myogenesis involves alterations in cell morphology, cell-cell contact, cell-extracellular matrix interactions, cell adhesion, and actin cytoskeleton dynamics [32]. Similarly, epithelial-mesenchymal transition (EMT), another highly enriched pathway in tumors with high-*GABRB3* expression, involves the loss of epithelial cell-cell adhesion, rendering them more motile and invasive, while acquiring a mesenchymal phenotype to invade distant organs and tissues [33]. The cytokines IFNG and IFNA can exert both pro-tumorigenic and anti-tumorigenic effects depending on their concentrations and the tumor microenvironment. In xenograft mouse models, IFNA exhibited both anti-proliferative and anti-metastatic effects in prostate cancer, potentially linked to an increase in the E-cadherin to matrix metalloprotease-9 ratio [34]. Clinical trials exploring the use of IFNA in prostate cancer treatment have encountered challenges owing to its high toxicity and limited efficacy [35]. Furthermore, in mouse models of metastatic prostate cancer, IFNG-treated xenografts exhibited significantly smaller tumor volumes than untreated counterparts [36]. However, a pro-metastatic role of IFNG has also been observed in prostate cancer cells, where it promotes EMT through the activation of Janus kinase/signal transducer and activator of transcription 1 signaling [37]. Additionally, IFNG induces the apoptosis of tumor-specific T cells, compromising antitumor immunity. Combination therapy with anti-CTLA-4 and anti-PD-1 antibodies can lead to excessive IFNG production, resulting in T-cell death and immune evasion [38]. These observations suggest that high-*GABRB3* expression have survival advantages, exhibit EMT, and display immune evasion phenotypes that promote prostate tumorigenesis and development.

The upregulation of *GABRB3* has been associated with the occurrence of brain metastases originating from diverse cancers, including prostate cancer [39]. A recent investigation revealed hypomethylation at the promoters of genes involved in neuroactive ligand-receptor interaction and cell adhesion molecules, such as *GABRB3*, in prostate cancer brain metastases. This observation suggests that cells from primary tumors may require specific reprogramming to facilitate the formation of brain metastases [40]. Stimulation of GABRB3 by GABA can activate the PI3K/AKT signaling pathways, which play a central signal to regulate cell survival, anti-apoptotic, and proliferation pathways [41]. The PI3K/AKT pathway is frequently hyperactivated in prostate cancer due to mutations or loss of PTEN, a tumor suppressor gene that inhibits PI3K. The hyperactivation of this pathway can compensate for the blockade of AR signaling, resulting in resistance to ADT and AR antagonists [42]. Our GSEA suggest that *GABRB3* plays varying roles in myogenesis, interferon γ and α responses, and the MYC proto-oncogene pathway during tumor development. Consequently, it is plausible that epigenetic dysregulation in primary prostate cancer leads to GABRB3 overexpression, subsequently activating the PI3K/AKT pathway and fostering resistance to ADT. However, further functional studies are warranted to validate our findings and elucidate the precise role of GABRB3 in the progression of prostate cancer.

Although our findings hold considerable promise, we acknowledge certain limitations. First, we conducted an analysis that focused solely on a subset of PI3K/AKT pathway genes. To gain a comprehensive understanding, future investigations should involve a thorough pathway analysis and fine mapping of causal variants. Second, the limited sample size and number of subgroups in our study may have limited the statistical power required to detect significant differences. Third, the homogeneity of our study population, consisting mainly of Taiwanese individuals, may have restricted the generalizability of our findings to diverse ethnic groups. Finally, while we present in silico functional evidence shedding light on the potential influence of GABRB3 on prostate cancer progression, further research is required to unravel the underlying mechanisms.

## Conclusion

This study revealed an intriguing link between GABRB3 and the survival of patients with prostate cancer undergoing ADT. The intricate interplay between multiple signaling pathways suggests a complex landscape during prostate cancer progression that requires comprehensive exploration. A deeper understanding of survival-related SNPs in the PI3K/AKT-related pathway may pave the way for more effective and personalized therapeutic strategies for prostate cancer management.

## Data Availability

The data that support the findings of this study are available from the corresponding author upon reasonable request.
